# Does the core circadian clock in the moss *Physcomitrella patens *(Bryophyta) comprise a single loop?

**DOI:** 10.1186/1471-2229-10-109

**Published:** 2010-06-15

**Authors:** Karl Holm, Thomas Källman, Niclas Gyllenstrand, Harald Hedman, Ulf Lagercrantz

**Affiliations:** 1 Program in Evolutionary Functional Genomics, Evolutionary Biology Centre, Uppsala University, Norbyvägen 18D, SE-752 36 Uppsala, Sweden; 2 Department of Plant Biology and Forest Genetics, Swedish University of Agricultural Sciences, SE-750 07 Uppsala, Sweden

## Abstract

**Background:**

The endogenous circadian clock allows the organism to synchronize processes both to daily and seasonal changes. In plants, many metabolic processes such as photosynthesis, as well as photoperiodic responses, are under the control of a circadian clock. Comparative studies with the moss *Physcomitrella patens *provide the opportunity to study many aspects of land plant evolution. Here we present a comparative overview of clock-associated components and the circadian network in the moss *P. patens*.

**Results:**

The moss *P. patens *has a set of conserved circadian core components that share genetic relationship and gene expression patterns with clock genes of vascular plants. These genes include Myb-like transcription factors *PpCCA1a *and *PpCCA1b*, pseudo-response regulators *PpPRR1-4*, and regulatory elements *PpELF3*, *PpLUX *and possibly *PpELF4*. However, the moss lacks homologs of *AtTOC1*, *AtGI *and the *AtZTL*-family of genes, which can be found in all vascular plants studied here. These three genes constitute essential components of two of the three integrated feed-back loops in the current model of the Arabidopsis circadian clock mechanism. Consequently, our results suggest instead a single loop circadian clock in the moss. Possibly as a result of this, temperature compensation of core clock gene expression appears to be decreased in *P. patens.*

**Conclusions:**

This study is the first comparative overview of the circadian clock mechanism in a basal land plant, the moss *P. patens*. Our results indicate that the moss clock mechanism may represent an ancestral state in contrast to the more complex and partly duplicated structure of subsequent land plants. These findings may provide insights into the understanding of the evolution of circadian network topology.

## Background

The importance of timing of biological processes to the day-night cycle is reflected in the ubiquity and independent evolution of circadian clock mechanisms in different taxa [1]. The endogenous circadian clock allows the organism to synchronize processes both to daily and seasonal changes. In plants, many processes such as photosynthesis that exhibit a daily rhythm are under the control of a circadian clock. The circadian system is also important to track seasonal changes, e.g. the anticipation of spring or autumn to induce reproduction [2]. Even though circadian clocks in bacteria, fungi, plants and animals have evolved largely independently and thus are composed of different sets of genes, these clocks show a common structure or underlying principle. Recent experimental data and modeling suggests that circadian systems are complex networks of several interlocked feedback loops and interconnected input and output circuits [3,4]. These loops contain both positive and negative elements on transcriptional and post transcriptional levels [5,4].

*Arabidopsis thaliana *(Arabidopsis) has been the main plant model in studies of plant circadian clocks, and the first identified feedback loop contained two closely related and partly redundant Myb-like transcription factors CIRCADIAN CLOCK ASSOCIATED 1 (CCA1) and LATE ELONGATED HYPOCOTYL (LHY) forming a negative feedback loop with TIMING OF CAB EXPRESSION 1 (TOC1 also referred to as PRR1) [6]. Experimental data have since shown that additional loops are needed to explain observed data. Mathematical modeling and empirical studies suggest that CCA1/LHY also form a second loop with PSEUDO RESPONSE REGULATOR 7 (PRR7) and PRR9 which both belong to the same family as TOC1. In addition, an evening phased loop is predicted including TOC1 and a factor that has been suggested to be GIGANTEA (GI) [7-9].

CCA1 and LHY are important regulators of circadian rhythm in Arabidopsis, not only involved in regulating the expression of *TOC1*, but also in mediating light input to the core oscillator [10-12]. CCA1/LHY regulate several output genes of the circadian clock by binding to the evening element of the promoter of these genes [13].

TOC1, or PRR1, share domains with the other PRR proteins (PRR9/7/5/3). The pseudo receiver domain is similar to the phospho-accepting domain found in authentic response regulators, and the plant specific CCT motif (CO, COL and TOC1) contains a putative nuclear localization signal and has also been shown to mediate protein-protein interactions [14,15]. PRR9, PRR7 and PRR5 have, besides regulatory roles close to the Arabidopsis core oscillator, also functions in the response of the clock to both light and temperature [16-18].

Simulations and experimental data suggest that GI is a component of the circadian core oscillator with rhythm defect phenotypes of mutants in both continuous light (LL) and continuous dark (DD) [19,20]. GI also has an important role in the photoperiodic control of flowering as it induces the circadian expression of *CONSTANS *(*CO*) in the late afternoon during long days in Arabidopsis [20]. In addition, GI has a function in temperature compensation of the circadian clock as a functional GI has been shown to extend the range of temperatures at which robust rhythmicity can be maintained [21].

Further components associated with core clock function in Arabidopsis include LUX ARRYTHMO (LUX), EARLY FLOWERING 4 (ELF4), EARLY FLOWERING 3 (ELF3), and members of the ZTL gene family, ZEITLUPE (ZTL), LOV KELCH PROTEIN 2 (LKP2) and FLAVIN-BINDING, KELCH REPEAT, F-BOX 1 (FKF1). ZTL has been shown to have a functional role in core clock function by controlling the cyclical degradation of TOC1 [22,23]. This is achieved by a blue light dependent protein interaction with GI, which stabilizes the ZTL protein [24]. LKP2 may have a similar role, although its effect on circadian clock function appears to be smaller [24,25]. FKF1 seems not to have a role in clock function itself but acts down stream of the core oscillator. The FKF1 protein is also stabilized by protein-protein interaction with GI, and regulates flowering time by targeted degradation of CDF2, a repressor of *CO *transcription [26-29]. Thus, all members of the ZTL family of proteins have functional roles closely associated to the evening phased loop comprising TOC1 and GI.

The Myb transcription factor LUX ARRHYTHMO, however, has been shown to interact with components of the morning-phased loop. While LUX is required for the expression of the core clock genes *LHY *and *CCA1*, the *LUX *promoter also contains an evening element, indicating that *LUX *itself is under negative control by LHY and CCA1 [30]. In Arabidopsis, ELF4 has been proposed to have a function in the core clock and recent experimental data suggest that ELF4 likely has two inputs, at both *PRR9*/*PRR7 *and *GI*/*LUX*, and functions to repress the light-induced expression of these components of both the morning- and evening-phased loops [31,32]. ELF3 acts in the gating of light input to the core oscillator and is necessary for light induced expression of *CCA1*/*LHY*; as such, ELF3 contributes to the resetting of the clock each day [33,34,32]. In addition, a recent study has demonstrated the dual role of ELF3 by showing that the protein regulates both circadian rhythm and flowering time in Arabidopsis by interacting with COP1 and thus mediating the cyclical degradation of GI [35]. Although a specific placement of ELF3 in the plant circadian network has not yet been determined, the ELF3 protein seems to interact with components of both the morning- and evening phased circuits of the current three-loop model.

Comparative studies of clock associated components and the circadian systems in photosynthesizing organisms have been carried out in the green algae *Chlamydomonas reinhardtii *and *Ostreococcus tauri *[36-38], and in several seed plants, *Mesembryanthemum crystallinum *[39], *Glycine max *[40], *Phaseolus vulgaris *[41], *Pisum sativum *[42], *Lemna gibba *and *Lemna paucicostata *[43,44], *Oryza sativa *[[45] and references therein], *Castanea sativa *[46,47], *Populus nigra *[48] and *Picea abies *[[49] and unpublished data, our lab]. The consensus result of studies in seed plants is that homologs to core clock genes in Arabidopsis are present and furthermore appear to display a high degree of functional conservation. However, the situation is different in the algae *C. reinhardtii *and *O. tauri*. Beside implicated similarities in the phototransduction (CRYPTOCHROMES) and kinase pathways (CKI and CKIIs), that constitute important input and regulatory components of the circadian clock, homology to core clock genes in Arabidopsis is limited to Myb-related and PRR family-like proteins [36-38] (Additional file 1).

As a representative of Bryophytes that separated from the lineage leading to vascular plants more than 400 MYA [50], *Physcomitrella patens *provides the opportunity to study many aspects of land plant evolution. Circadian rhythms and photoperiodic responses are known from all categories of plants from algae and mosses to higher plants. In *P. patens*, as in higher plants, photoperiod affects the switch from vegetative growth to reproduction, as short days (SD) induce sporophyte development in Physcomitrella [51]. Diurnal rhythms in expression have also been observed for some *P. patens *genes involved in photosynthesis [52,53]. Furthermore, the presence of *CONSTANS*-like (*COL*) genes with a diurnal expression pattern has been reported [54,4]. These genes are related to the central regulator of flowering time, *CO *in Arabidopsis, but their involvement in photoperiodic induction of reproduction in *P. patens *is not supported by available data [[54,55]; our own unpublished data]. Finally, two homologs to known circadian clock genes in higher plants, *PpCCA1a *and *PpCCA1b*, have recently been characterized in *P. patens *[56].

To systematically identify putative photoperiod pathway and circadian clock components in *P. patens *and to study the evolution of the circadian clock, genes associated with the photoperiod pathway in Arabidopsis (Additional file 1) were used in BLAST searches against the genome sequence of selected plant species representing algae, non-vascular plants, non-seed vascular plants as well as seed plants. Identified putative *P. patens *clock genes were further characterized by phylogenetic analyses and by assessing their temporal expression profiles in photoperiod and free-running conditions.

## Results

Database searches in sequenced genomes representing algae, mosses, lycophytes, and angiosperms identified a limited set of homologs to genes implicated in circadian clock in non-land plants, while homologs to a majority of the included Arabidopsis clock associated genes were identified in all land plants (Additional file 1), with a few striking exceptions in moss (see below). These data suggest that a majority of the components in the circadian clock present in higher plants arose with the evolution of land plants. To study the evolution of the circadian clock of land plants in more detail, we focused on *P. patens *representing the earliest land plant with a sequenced genome, with the aim of identifying the putative core circadian clock genes present early in the evolution land plants. We focused on a set of genes for which there is strong evidence for a circadian clock function in higher plants.

All studies of diurnal and circadian expression of Physcomitrella genes have thus far observed clear cycling under light-dark cycles (LD) and/or in DD, but rapid dampening of the rhythm in LL [[52-56]; our own unpublished data]. This is in contrast to the situation in Arabidopsis, where endogenous expression rhythms can more readily be measured in LL whereas they dampen more quickly in DD [14,40,31,34]. It is perhaps premature to state that the circadian clock in Physcomitrella is dysfunctional in LL, as has been suggested [56], and more studies of clock behavior in LL are needed. However, in the present study we chose to concentrate on rhythmic expression under LD and DD conditions.

### Putative clock genes in *P. patens*

#### Single Myb domain genes related to *CCA1 *and *LHY*

**Figure 1:**
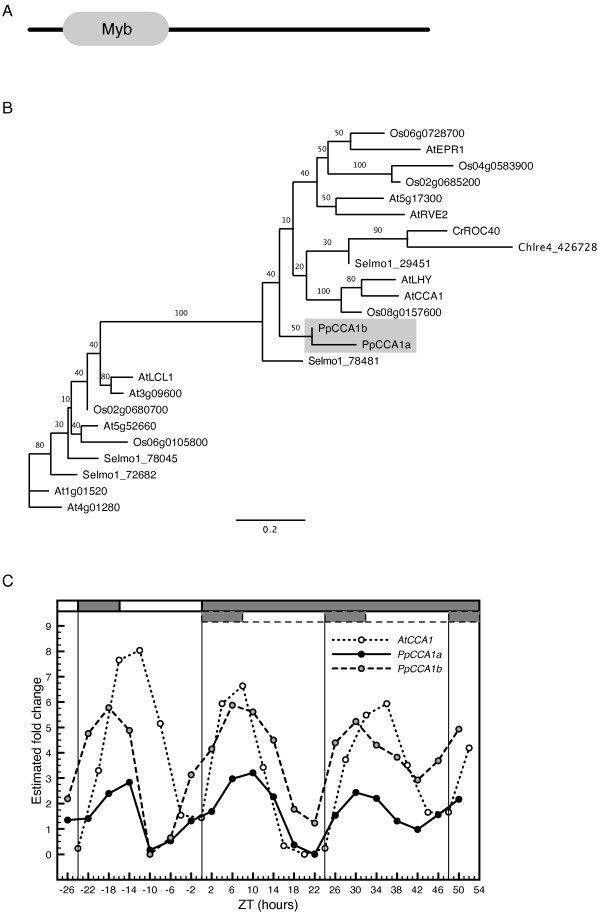
**Characterization of putative *CCA1/LHY *orthologs in *P. patens***. Characterization of putative CCA1/LHY orthologs in *P. patens*. (A) Domain structure of CCA1 and LHY in *Arabidopsis *with the single Myb-domain. (B) An unrooted maximum-likelihood phylogenetic tree constructed from an alignment of the Myb-domain of the SHAQKYF subtype in protein sequences found in *A. thaliana*, *O. sativa*, *S. moellendorffii*, *C. reinhardtii *and *P. patens*. (C) Comparative analysis of the oscillating profiles of *AtCCA1*, *PpCCA1a *and *PpCCA1b *in *A. thaliana *and *P. patens*. *P. patens *cultures were sampled every fourth hour for one day in LD (16 h light/8 h dark) and for two days in DD (constant dark). Comparative data for *AtCCA1 *was downloaded from the DIURNAL database (see Methods). Quantitative RT-PCR expression data was normalized (CT_target _- CT_reference_) and the minimum transcription level in each time series was arbitrarily set to 0. Grey and white bars surrounded by dashed lines indicate subjective night and day in DD. Plots including error bars indicating standard deviation from duplicate runs of all genes can be found in Additional file 6.

The transcription factors CCA1 and LHY contain a single Myb-like domain of the well-conserved SHAQKYF motif at the N-terminal [Figure 1A, [57]]. One ortholog to CCA1/LHY has been identified and characterized in *O. sativa *[45] and we identified two predicted proteins in *Selaginella moellendorffii *that share significant sequence homology to CCA1/LHY (Figure 1B). Two predicted protein sequences containing related single Myb domains were also found in *C. reinhardtii*. The DNA-binding domain in Chlre4-402780 was identical to the Myb domain in the previously described ROC40 [37]. A putative CCA1 homolog has also recently been identified and characterized in *O. tauri *[38]. In *P. patens*, two orthologs have been identified and characterized, *PpCCA1a *and *PpCCA1b *[58,56]. In the present study, *PpCCA1a *and *PpCCA1b *show stable circadian expression patterns in LD and for 48 hrs in DD without any apparent dampening (Figure 1C). The rhythmic pattern was statistically significant with COSOPT *pMMC-β *values at 0.026 for *PpCCA1a *and 0.030 for *PpCCA1b *(a listing of all statistical test results can be found in Additional file 2). In addition, phases are well superimposed onto those of *AtCCA1 *and *AtLHY *with peaks close to dawn.

#### Pseudo-response regulators (PRR)

**Figure 2:**
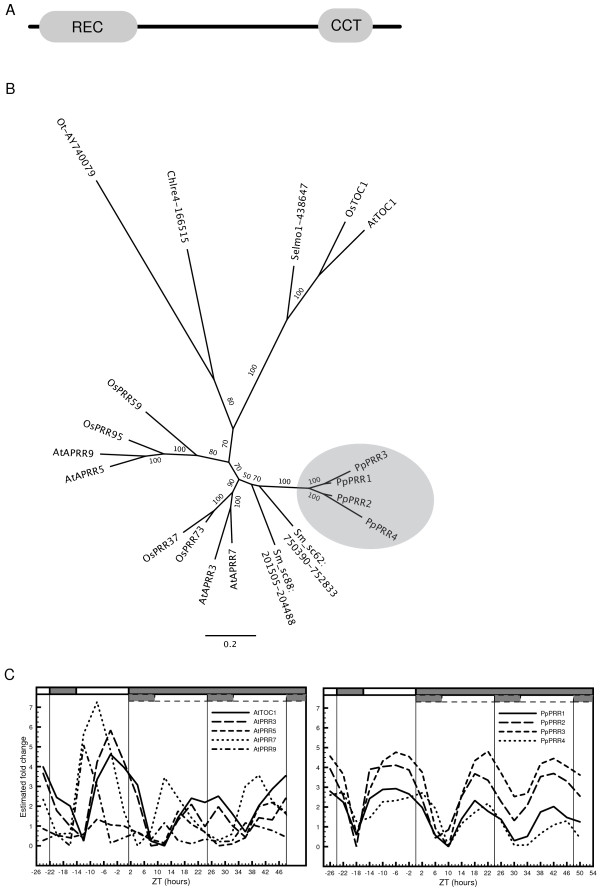
**Characterization of putative *PRR *orthologs in *P. patens***. Characterization of putative PRR orthologs in *P. patens*. (A) Domain structure of TOC1 in *Arabidopsis *with the pseudo-receiver domain at the N-terminal and CCT motif at the C-terminal. (B) An unrooted maximum-likelihood phylogenetic tree was constructed from the amino acid sequences of the pseudo-receiver and the CCT motif in *A. thaliana*, *O. sativa*, *S. moellendorffii*, *C. reinhardtii, O. tauri *and *P. patens*. (C) Comparative analysis of the expression profiles of *TOC1 *and the four *PRRs *in Arabidopsis (left panel), and the expression profiles of *PpPRR1-4 *in *P. patens *(right panel). See legend to figure 1 for further details.

The receiver domain at the N-terminal of the pseudo-response regulators, TOC1/PRR1 and PRR9/7/5/3, differ from the authentic response regulators, the ARR-family, by lacking an aspartate at the phospo-accepting site of the domain [59]. In addition, the PRRs include a CCT motif at the C-terminal of the polypeptide (Figure 2A). Orthologs to TOC1 and the PRR family of proteins have been identified and extensively characterized in *O. sativa *[60,45]. One *TOC1*-like sequence, Selmo1_438647, and its allelic variant, Selmo1_447266 (not shown in tree 2B), are annotated as predicted proteins in the Selaginella genome database, although both gene annotations contain a stop codon before the C-terminal CCT domain. Four additional *S. moellendorffii *sequences positioned on scaffold_88:201505-204488 and scaffold_62:750390-752881, including their probable allelic variants located on scaffold_42:1313168-13161115 and scaffold_20:1992667-1995113, cluster within the remaining PRR sequences (Figure 2B). These are not annotated as predicted proteins and lack protein ID numbers, because they appear as fused together with other genes. Even if it cannot be determined at present whether these putative proteins are expressed and translated, it appears obvious that the corresponding nucleotide sequences are present in the *S. moellendorffii *genome. In version 4.0 of the *Chlamydomonas *genome database, only one predicted amino acid sequence displays resemblance to the domain structure of the TOC1/PRR family of proteins, Chlre4-166515 [Additional file 1]. This protein clusters with a recently identified and described PRR-like protein in another unicellular green alga, *Ostreococcus tauri *[38]. This protein, Ot-AY740079, also shares domain structure with the TOC1/PRR family of proteins and has a clearly shown function within the *O. tauri *circadian clock [38]. However, although the *O. tauri *protein is referred to as a TOC1-homologue, the phylogenetic analysis reveals a distant relationship to the remaining TOC1/PRR family of proteins (Figure 2B). This suggests that the algal proteins constitute sister lineages to the whole TOC1/PRR family, and that it is not possible from presently available data to determine whether these proteins are more closely related to the TOC1-group, or the group consisting of the remaining PRR proteins. We therefore choose to refer to them as PRR family-like (Additional file 1). Four pseudo-response regulator homologs have been identified in *P. patens*, *Pp*PRR1-4 [Figure 2B, [58]]. *Pp*PRR1 to *Pp*PRR4 are closely related and form a cluster next to the *S. moellendorffii *PRRs in the phylogenetic tree (Figure 2B). Thus, no ortholog to *TOC1 *appears to exist in *P. patens*. All putative response-regulators in *P. patens *show stable rhythm under LD conditions, with very slight dampening of amplitude in DD (COSOPT pMMC-β values ranging from 0.019 to 0.049) (Figure 2C, right panel). However, under our sampling scheme, we could not detect any differential expression of phase peaks among *PpPRR1 *to *PpPRR4*, similar to the sequential expression of phase displayed by the Arabidopsis quintet of *PRR *genes [Figure 2C, left panel; [60]]. Rather, our data suggest that the closely related, and probably recently duplicated *PpPRR1 *to *PpPRR4*, have not diverged in terms of their temporal gene expression pattern.

The emergence in vascular plants of a markedly diverged *TOC1 *sub-family distinct from the other *PRR *genes, suggests that positive Darwinian selection might have been important in the evolution of *TOC1 *genes. To test this hypothesis, we estimated dN/dS ratios in a phylogenetic tree including identified *PRR *and *TOC1*-like genes from a diverse set of species (Additional file 3). The results suggest that the branch leading to the *TOC1 *clade has diverged due to adaptive evolution (*2Δl *= 6.75, *p *< 0.01) and 21.8% of the sites in the two conserved domains show a significant signal of positive selection (Additional file 3).

#### GIGANTEA

In Arabidopsis, GI is a 127-kD nuclear protein consisting of 1173 amino acids. It does not contain any previously described protein domains or motifs. Orthologs to *GI *have been identified in *O. sativa *[61], and in addition, we found two predicted protein sequences in *S. moellendorffii*, Selmo1-140066 and Selmo1-170553, that displayed significant sequence homology to AtGI (Additional file 1). However, *GI *appear to be absent in *Chlamydomonas reinhardtii *and in *O. tauri *as previously reported [Additional file 1, [36-38]].

Similarly, no searches for *GI*, on either nucleotide or protein levels, yielded any hits against version 1.1 of the *P. patens *genome database. We conclude that *GI*, or any *GI*-like sequences, are absent in the Physcomitrella genome.

#### LUX ARRYTHMO

**Figure 3:**
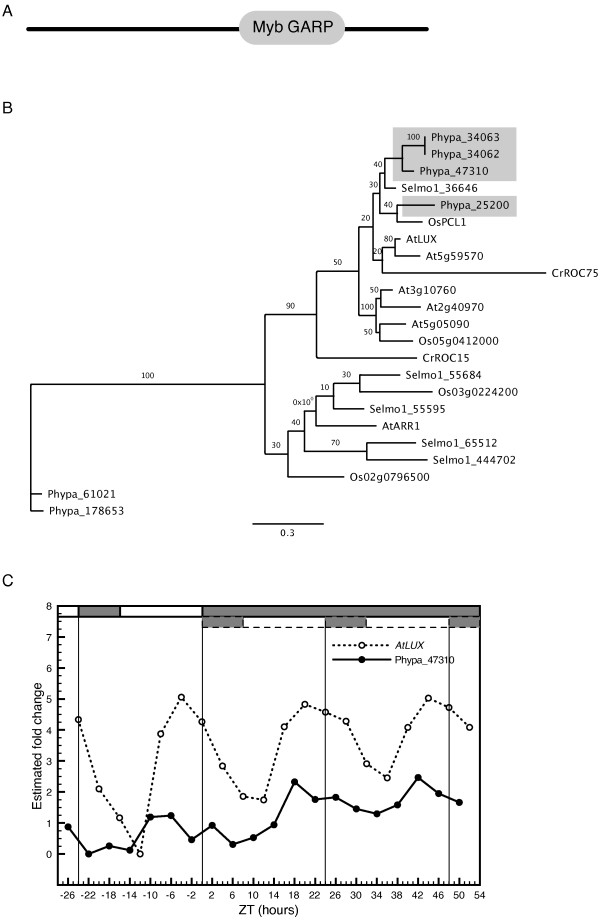
**Identification and characterization of putative *LUX *orthologs in *P. patens***. Identification and characterization of putative LUX orthologs in *P. patens*. (A) Domain structure of LUX in *Arabidopsis *with the Myb DNA binding motif toward the C-terminal. (B) An unrooted maximum-likelihood phylogenetic tree was constructed from an alignment of the Myb domain in *A. thaliana*, *O. sativa*, *S. moellendorffii*, *C. reinhardtii *and *P. patens*. (C) Comparative analysis of the expression profile of *LUX *in Arabidopsis and the putative ortholog *Phypa-47310 *in *P. patens*. See legend to figure 1 for further details.

LUX ARRYTHMO (LUX) or PHYTOCLOCK 1 (PCL), contains a Myb-related DNA-binding domain similar to, but distinct from those found in LHY and CCA1 [Figure 3A; [30,62]]. A *LUX *ortholog has been identified in *O. sativa *[45] and one predicted protein in *S. moellendorffii *displays sequence homology to AtLUX (Figure 3B). Two proteins in *C. reinhardtii*, CrROC15 and CrROC75, also include Myb DNA binding motifs that share homology with AtLUX [37]. A search for putative *P. patens *orthologs revealed four predicted protein sequences that clustered together with OsPCL1 and AtLUX (Figure 3B). Of these putative *LUX *orthologs, only *Phypa-47310 *showed a tendency to a circadian expression pattern (Figure 3C). Although the rhythmic expression pattern of *Phypa-47310 *was not significant (COSOPT *pMMC-β *= 0.23 and Fischer's exact g *p*-value = 0.07), a visual inspection of the expression pattern in DD suggests a periodic cycling with a phase peak very similar to that of *AtLUX *(Figure 3C).

#### EARLY FLOWERING 4

**Figure 4:**
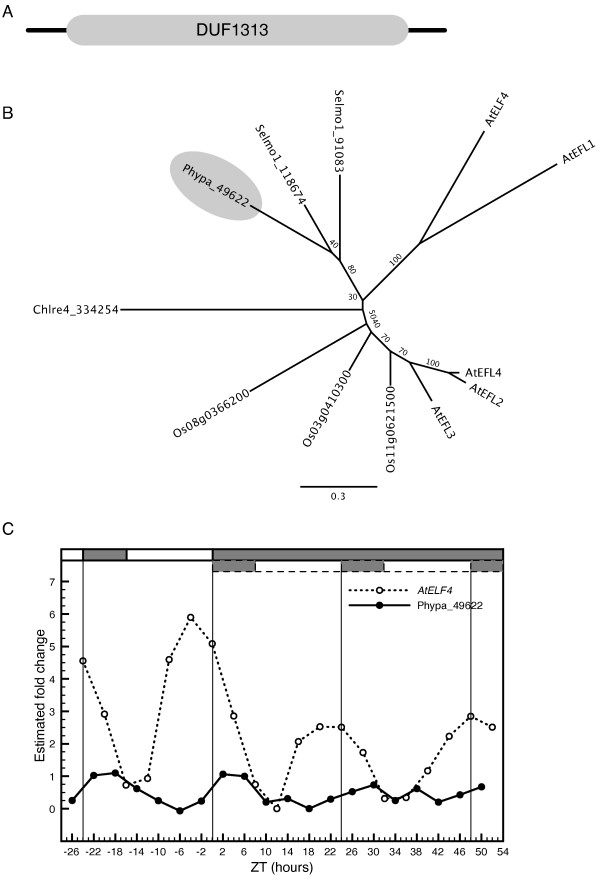
**Identification and characterization of a putative *ELF4 *ortholog in *P. patens***. Identification and characterization of a putative ELF4 ortholog in *P. patens*. (A) Domain structure of ELF4 in *Arabidopsis *with the DUF1313 domain covering most of the amino acid sequence. (B) An unrooted maximum-likelihood phylogenetic tree was constructed from an alignment of the DUF1313 region in *A. thaliana*, *O. sativa*, *S. moellendorffii, C. reinhardtii *and *P. patens*. *AtEFL1 *is At2g29950, *AtEFL2 *is At1g72630, *AtEFL3 *is At2g06255 and *AtEFL4 *is At1g17455. (C) Comparative analysis of the expression profile of *ELF4 *in Arabidopsis and the putative ortholog *Phypa-49622 *in *P. patens*. See legend to figure 1 for further details.

ELF4 belongs to the DUF1313 family of plant proteins; a group of relatively short proteins with unknown function (Figure 4A). Several gene products in both *O. sativa *and *S. moellendorffii *show significant sequence homology to AtELF4 (Figure 4B); however, the expression patterns of the rice sequences were reported not to be under circadian control and thus not considered to be ELF4 orthologs [45]. A predicted protein incorporating the DUF1313 domain is also present in *C. reinhardtii *[Additional file 1]. In our phylogenetic analysis of the DUF1313 protein sequences, AtELF4 clusters together with AtEFL1, while AtEFL2-4 form a separate and more distantly related lineage (Figure 4B). This grouping is supported by recent detailed analyses of the Arabidopsis DUF1313 proteins, where in addition, it was shown that only *AtEFL1 *were able to fully complement the *elf4 *loss-of-function phenotype [32]. One predicted protein in *P. patens*, Phypa-49622, displays significant sequence homology to the DUF1313 domain and clusters together with the *Selaginella *sequences (Figure 4B). However, the expression profile for *Phypa-49622 *displayed very weak amplitude in DD and was out of phase with *ELF4 *in Arabidopsis (Figure 4C). Present data suggest that *Phypa-49622 *is not likely to be a functional homolog of *AtELF4*.

#### EARLY FLOWERING 3

**Figure 5:**
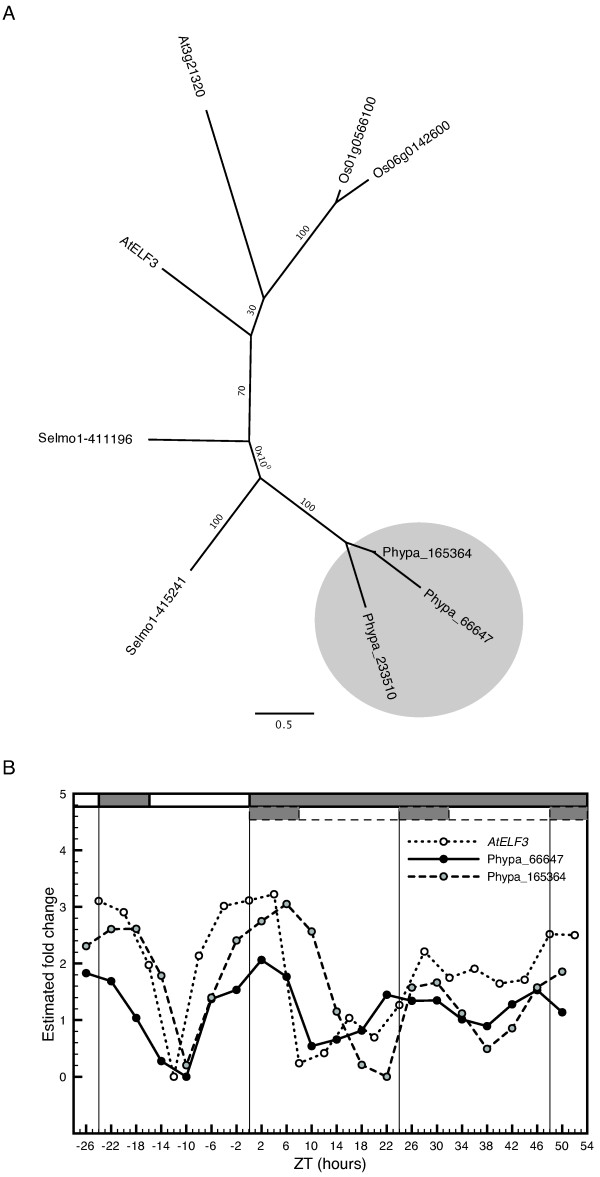
**Identification and characterization of putative *ELF3 *orthologs in *P. patens***. Identification and characterization of putative ELF3 orthologs in *P. patens*. (A) An unrooted maximum-likelihood phylogenetic tree was constructed from an alignment of conserved regions of ELF3-like amino acid sequences in *A. thaliana*, *O. sativa*, *S. moellendorffii *and *P. patens*. (B) Comparative analysis of the expression profile of *ELF3 *in *A. thaliana *and the two putative orthologs, *Phypa-66647 *and *Phypa-165364*, in *P. patens*. *Phypa-233510 *did not display a rhythmic expression pattern (data not shown). See legend to figure 1 for further details.

The ELF3 protein does not contain any known functional domains or motifs. Two gene products in *O. sativa*, Os01g0566100 and Os06g01426000, show significant sequence homology to AtELF3, however, their genes do not display any circadian expression pattern in rice [45]. We found two sequences in *S. moellendorffii *with significant homology to ELF3, Selmo1-415241 and Selmo1-411196 including their allelic variants Selmo1-443557 and Selmo1-443909, respectively (not shown in figure 5). No ELF3 like proteins were found in the latest version of the *C. reinhardtii *database [36]. A search of the *P. patens *database produced three predicted proteins with significant sequence homology to AtELF3, that all cluster together in the phylogenetic tree (Figure 5A). Of the three putative *ELF3*-like genes, *Phypa-66647 *and *Phypa-165364 *displayed an oscillating pattern in both LD and DD (COSOPT *pMMC-β *values = 0.025 and 0.16, respectively), with a peak of phase that agreed well with expression data of *ELF3 *in Arabidopsis (Figure 5B).

#### The ZEITLUPE family

**Figure 6:**
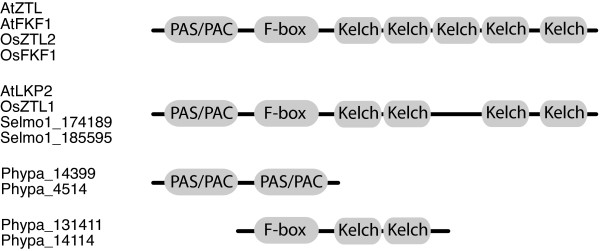
**Protein architecture of the *ZTL*-family of genes**. Protein architecture of ZTL family-like genes in *A. thaliana*, *O. sativa*, *S. moellendorffii *and *P. patens.*

ZTL, and its homologs LKP2 and FKF1, are unique F-box proteins with a PAS/PAC domain at the N-terminal and a series of Kelch repeats at the C-terminal [26,25,63]. The PAS/PAC domain is a blue light receptor also found in PHOTOTROPINS, whereas the F-box mediates ubiquitination of specific target proteins [64]. The Kelch repeats form groups of beta sheets that interact with other proteins [65]. Six separate Kelch-repeats have been identified in AtZTL and AtFKF1 [26,25,63]; however, for comparison we display only five repeats in figure 6, which is the result obtained from the SMART database [95,96]. Three proteins with domain structures similar to the ZTL family in Arabidopsis have been identified in *O. sativa*, *Os*ZTL1, *Os*ZTL2 and *Os*FKF1 [45]. Two predicted proteins in *S. moellendorffii *with high sequence similarity, Selmo1-174189 and Selmo1-185595, also share this typical domain structure. In accordance with previous studies, we could not detect any ZTL family-like proteins or predicted protein sequences in *Chlamydomonas reinhardtii *or *O. tauri *[Additional file 1, [36-38]]. Our scan of the *Physcomitrella *genome revealed several proteins containing the above described functional domains, however, the three domains were never present in the same predicted protein sequence. Instead, the PAS/PAC domain was found in duplicates in several predicted phototropins, e.g. *Pp*PHOTA1, *Pp*PHOTB1 and *Pp*PHOTB2. The duplicate PAS/PAC domains also occurred in isolation, as in Phypa-143199 and Phypa-4514 (Figure 6). F-boxes followed by Kelch-like repeats could be found in two predicted proteins in *P. patens*, Phypa-131411 and Phypa-14114 but never with the PAS/PAC domain at the N-terminal (Figure 6). Thus, proteins with a domain architecture analogous to the one found in the Arabidopsis ZTL family, in three annotated *O. sativa *proteins and in two predicted *S. moellendorffii *protein sequences, appear to be absent in *P. patens*.

### Temperature compensation in *P. patens*

**Figure 7:**
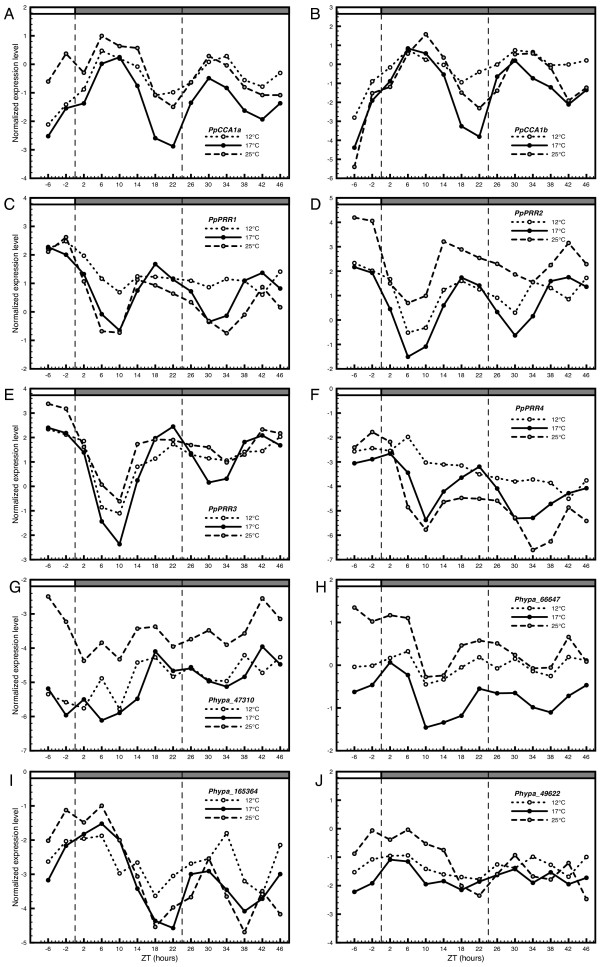
**Time series expression pattern of putative clock genes in different temperatures**. Normalized expression levels (CT_target _- CT_reference_) for putative circadian clock genes in *P. patens *measured under three different temperatures in DD (constant dark). The light regime was identical to previous experiments (see legend to figure 1 for further details). Temperature was changed one day (ZT-24) before the onset of DD.

GI has been implicated in QTL mapping experiments and mutant studies as a strong candidate for mediating temperature compensation in the Arabidopsis circadian clock [66,21]. It has been shown that GI critically affects clock function under natural conditions by extending the range of temperatures at which circadian rhythmicity can be maintained and period length remains stable. In particular, it would appear that the GI/TOC1 loop participates by regulating the expression levels of *CCA1 *and *LHY *at both low (12°C) and high (27°C) temperatures [21]. Since neither GI, nor a GI/TOC1 loop appear to be present in *P. patens*, it could be hypothesized that the ability to compensate for changes in ambient temperature is either maintained by other components related to the clock mechanism, or is reduced in the moss. To test the temperature response of putative clock genes in *P. patens*, cultures were sampled in parallel in constant dark (DD) in three different temperatures, 12°C, 17°C and 25°C. Temperature was changed one day before the onset of DD at ZT -24. Gene expression levels were measured for all putative clock genes in *P. patens *(Figure 7A-J).

A decrease in amplitude and less well defined phase peaks are evident in the expression pattern of all genes at lower and higher temperatures. The broader phase peaks are noticeable as early as the first day in DD, whereas the loss of amplitude is more evident during the second day. These indications of a less robust rhythmic pattern are more pronounced at 12°C where several genes lose any trace of rhythmic expression pattern (Figure 7C-E, H). Although a slight decrease in amplitude can also be seen during the second day at 17°C, most likely due to a lack of photoperiodic input, most genes maintain a well-defined phase peak at this temperature. The observed decay of robust rhythm is reflected in the statistical analysis of the time series data. At 17°C, expression patterns of *PpCCA1a-b*, *PpPRR1-4 *and *PpELF3L1 *all display a significant circadian rhythm as reported by COSOPT (*pMMC-β *values ≤ 0.05) (Additional file 2). The group of significantly oscillating genes are reduced to *PpCCA1a-b *and *PpPRR1-2 *at 25°C, whereas only *PpCCA1a-b *maintain significant rhythms at 12°C, as reported by either COSOPT or Fisher's exact G test (Additional file 2). Although estimates of period length reported by COSOPT are not to be taken at face value under the present sampling scheme, it is notable that all significant expression patterns cluster together at 17°C with an estimated period length of approximately 22 h (Additional file 4). In summary, time series expression data suggest that the temperature interval of robust circadian rhythm is more narrow in *P. patens *compared to wild type Arabidopsis. However, the comparatively robust rhythm and stable period lengths of *PpCCA1a *and *PpCCA1b *at all temperatures may indicate a degree of temperature compensation of these core components independent of GI or a GI/TOC1 loop.

## Discussion

The moss, *P. patens*, has a set of conserved clock-associated components, which share genetic relationship and gene expression profiles with clock genes of higher plants. These components include members of the Myb family of transcription factors, *Pp*CCA1a and *Pp*CCA1b, and four pseudo-response regulators, *Pp*PRR1-4 [58,56]. Further putative clock genes present in the moss include one LUX-like, as well as two ELF3 and possibly one ELF4-like component of the Arabidopsis circadian mechanism (see Additional file 1 for a comparative overview of clock associated genes in Arabidopsis and *P. patens)*. However, we note that *P. patens *appears to lack any genes coding for proteins orthologous to TOC1, GI or any of the ZTL family of proteins, all of which are present in *S. moellendoerffii*. Genes orthologous to some or all of these genes have also been reported in a number of seed plants, e.g. the monocotyledons rice [61,45], two species of *Lemna *[43,44], the angiosperms soybean [40], pea [42], chestnut [46], and the gymnosperm Norway spruce [49].

None of the three core clock genes absent in the moss are present in the green algae *C. reinhardtii*, as has been previously reported [36,37] or *O. tauri *[38], if as we propose, *OtTOC1 *constitute a sister lineage to the whole PRR gene-family rather than a *TOC1 *ortholog. The result of the phylogenetic analysis of the pseudo-response regulator family of proteins implies that a common ancestor of all *PRR *genes was likely present already in the green algae *C. reinhardtii *and *O. tauri *[Figure 2B and Additional file 1, [37,38]]. This notion seems further supported by the fact that the *OtPRR*-like sequence is the only pseudo-response regulator to date that display a functional phosphate acceptor aspartyl residue, implying recent divergence from the ARR family of proteins [38]. These data collectively suggests that the three core clock genes have not been lost in the moss, but rather appeared before, or simultaneously, with the emergence of the vascular plants.

The four PRR proteins in *P. patens *display a high degree of sequence similarity and cluster tightly together among the PRR proteins of higher plants, but are as a group well separated from the branch including the TOC1/PRR1s of these plants (Figure 2B). The relatively recent expansion of the *P. patens PRR *gene family is reflected in the expression profiles of *PpPRR1-4*; while the *Arabidopsis PRR9/7/3/5 *and *AtTOC1 *display a serial distribution of phase peaks suggesting functional divergence [17], the *PRR *orthologs in *P. patens *have a very similar expression pattern with near identical phase peaks (Figure 2C).

Our data suggest that the TOC1/PRR1 orthologs in *S. moellendorffii*, *O. sativa *and Arabidopsis represent a more recent and functionally divergent variant among the two component pseudo-response regulators. A functional divergence of the *TOC1*-like genes compared the other *PRR *genes is supported by the elevated dN/dS ratio inferred on the branch separating the *PRR *and *TOC1 *genes. It suggests that a *PRR *gene duplication in the lineage leading to present day vascular plants was followed by positive selection on one of the copies possibly due the recruitment of new genes and features to the circadian clock. It is tempting to speculate that the adaptive evolution of *TOC1 *is coupled with the occurrence of GI and ZTL with which TOC1 closely interact.

While proteins with a domain architecture analogous to the ZTL family of proteins are also absent in the moss, the functional domains of these proteins do appear in other combinations; these sequences include predicted phototropins and also F-box proteins followed by Kelch repeats, but without the LOV domain at the N-terminal (Figure 6). From sequence homology alone, we have not been able to determine if any of these domains are more closely related to corresponding domains of ZTL like proteins in higher plants. In any case, the emergence of ZTL family-like proteins in *S. moellendorffii *suggest the formation of this novel protein architecture from the modular assembly of already existing functional domains, possibly through mechanisms such as duplication followed by domain shuffling [67,68].

No trace of *GI *or GI-like sequences can be found at the nucleotide or protein levels in available complete genomes other than in *S. moellendorffii *and in representatives of higher plants (see references above). Although gene duplication is considered one of the most common and important mechanisms for the emergence of novel genes, it is also known that each genome harbors a certain set of genes that cannot be associated with other known genes and that often are of unclear origin [69]. Occasionally, these orphan, or *de novo*, genes can be traced to ancestral noncoding DNA and intergenic regions [70-72], but there are also instances, e.g. in *Drosophila *and rice, where the exact origin of functional genes remains obscure [73,74]. It may be that the additional sequencing of full genomes representing different phylogenetic lineages may help to reveal an origin of *GI *in intergenic regions of early land plants or algae. However, there is also the rare possibility of horizontal gene transfer, which has been exemplified as occurring between fungi and early plant lineages such as bryophytes and lycophytes [75]. Presently, it would appear that *GI *is a *de novo *gene with unclear origin in the land plant lineage.

**Figure 8:**
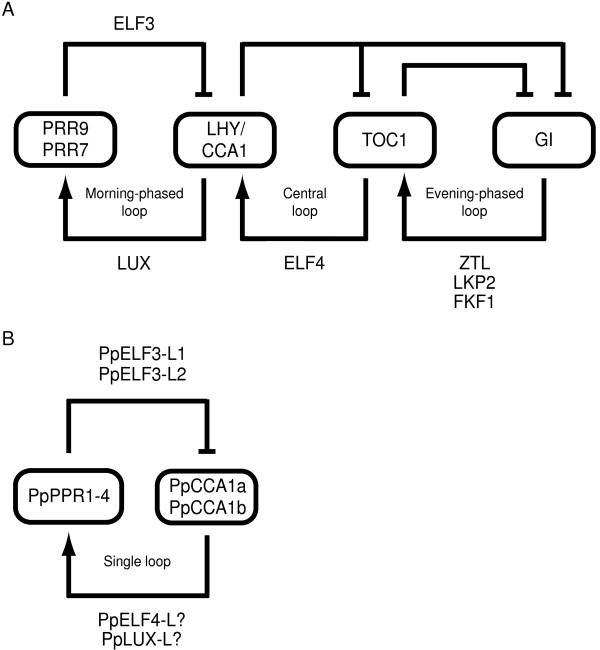
**Core clock network comparison**. Comparison of the core clock network in Arabidopsis and the version of the core clock in *P. patens *proposed in this study. (A) Locke's three-loop model where the inferred but as yet unidentified component "X" linking the feedback from TOC1 to LHY/CCA1 has been omitted for clarity. LUX has been placed in the vicinity of the morning-phased loop since interactions with LHY/CCA1 has been experimentally verified (see text for further details). Similarly, the ZTL family of components has been placed by the evening-phased loop. ELF4 has been positioned below the central loop since recent results suggest that it interacts with components of both the morning- and evening-phased loop (see text for further details). The positioning of ELF3 is relative since the exact means by which it interacts with the input light pathway, and feed back rhythmic information from the core clock, is not yet fully understood. (B) A suggested version of the *P. patens *circadian clock network. The single loop corresponds to the morning-phased loop of the Arabidopsis clock mechanism in seedlings and the "simplified slave version" of the Arabidopsis root clock. PpELF3L1-2 refer to Phypa_66647 and Phypa_165364. PpELF4L and PpLUXL refer to Phypa_49622 and Phypa_47310.

In view of the current three-loop model of the Arabidopsis circadian clock, the components absent in *P. patens*, TOC1 and GI, comprise an entire feed-back loop, also referred to as the evening-phased loop, or the evening oscillator [Figure 8A, 7]. In addition, TOC1 acts as the second feed-back component together with CCA1/LHY in the originally described central loop model of the Arabidopsis clock. Thus, while the clock mechanism of higher plants may at least comprise a three-loop design, our findings imply a single-loop for the moss clock (Figure 8B). It is notable that ZTL, LKP2 and FKF1, with known functions closely related to TOC1 and GI in Arabidopsis, also appear not to be present in *P. patens*. The singular morning-phased loop in the *P. patens *circadian mechanism would then consist of *Pp*CCA1a/*Pp*CCA1b joined together with one or some of the *Pp*PRRs present in the moss (Figure 8B). Interestingly, such a hypothesized single-loop plant clock is entirely analogous to the "simplified slave version" of the circadian clock described in Arabidopsis roots. In roots, the morning-phased loop is decoupled from the central- and evening-phased loops and the clock appears to run only on the feed-back interaction between CCA1/LHY and PRR9/7 [76]. However, in a recent study it was shown that double disruptants of *PpCCA1a *and *PpCCA1b *in the moss still display rhythmic output of the plastid sigma factor *PpSIG5 *and a member of the *PpPRR*-quartet [56]. This suggests the presence of further components, and/or possibly additional unknown loops, other than a TOC1/GI loop, that contribute to the maintenance of endogenous circadian rhythm in *P. patens*. Recent experimental data illustrate a similar situation in the Arabidopsis three-loop model, where triple mutants *prr9-10*, *prr7-11 *and *toc1-2*, still display detectable endogenous rhythm in *LHY/CCA1 *expression, although no feed-back loop should remain intact [77]. To what extent known clock components ELF3, ELF4 and LUX may contribute to this rhythm in Arabidopsis is still unknown.

In Arabidopsis, the protein GI has a critical effect on robustness of circadian rhythmicity and stability of period length of clock output during prolonged fluctuations in temperature [21]. Our assay of temperature response of putative clock genes in *P. patens *shows that most genes lose their circadian expression at temperatures 12°C and 25°C in constant dark (Figure 7). The decay of rhythmic expression pattern is most pronounced at 12°C where only *PpCCA1a *and *PpCCA1b *maintain significant circadian oscillation (Additional file 2 and 4). One reason for this reduced level of temperature compensation may be the absent GI/TOC1 loop in the moss.

The proposed simpler loop structure of the circadian clock in *P. patens *may have implications for the evolutionary origins and development of clock network topology. Hallmark features of endogenous circadian oscillators include the ability to maintain period length under constant conditions, to compensate for fluctuations in ambient variables, and yet be able to entrain, or reset, the clock daily according to environmental cues of e.g. light and temperature [78]. It has been pointed out that the complex loop structure of different circadian clock mechanisms may be a result of these inherently conflicting evolutionary aims of simultaneous robustness and sensitivity [79]. Mathematical modeling and simulation studies have shown that the degree of flexibility in an interconnected metabolic system, for example its ability to attain several aims simultaneously, depends less on the number of components in such a system, than on the structure of the network itself. Accordingly, there would be a selective advantage in increasing loop number and complexity, as well as on mechanisms that would enable this, e.g. gene duplication [79,80]. In this perspective, the circadian clock in *P. patens *may well represent an antecedent state comprising a single loop structure in contrast to the, in part, duplicated three-loop clock design of subsequent land plants.

## Conclusions

Although the Arabidopsis three-loop model has proven to have greater realism than previous models, and provides a valuable framework for comparative studies, it is not yet complete. It was initially pointed out that the model in its proposed version did not account for all known clock associated genes or interactions, and experimental data continues to imply the presence of unknown components and possibly additional loops [7,81,77]. We believe that the further study of a seemingly less complex network system not only can provide important insights into the evolution of circadian network topology, but in addition, may help clarifying some of the remaining issues of the circadian clock mechanism of higher model plants.

## Methods

### Plant materials and growth conditions

*Physcomitrella patens *of the Gransden Wood wild type strain was cultured as protonemal tissue on cellophane covered BCD medium (0.8% agar, 1 mM MgSO_4_, 1.85 mM KH_2_PO_4_, 10 mM KNO_3_, 45 μM FeSO_4_, 1 mM CaCl_2_, 1× Hoagland's Number 2 solution), supplemented with 5 mM ammonium tartrate. All cultures were grown in Sanyo MLR-350 growth chambers supplied with white fluorescent tubes, Toshiba FL40SS W/37 (430-650 nm), at 65-75 μmol m^-2 ^s^-1^. Light/dark (LD) conditions used were 16 h light/8 h dark, followed by constant dark (DD). Cultures were grown at 20°C, and transferred to temperatures 12°C, 17°C and 25°C one day before the onset of DD as noted in the text for each experiment. Cultures transferred to 17°C were sampled every 4th hour for 72 hours, the first day under LD conditions, followed by two days in DD. Cultures transferred to 12°C and 25°C were sampled every 4th hour for 52 hours, two time points in light followed by 12 time points in DD, as indicated in each figure.

### RNA sample preparation and cDNA synthesis

Total RNA was extracted from each time point sample using the RNeasy plant mini kit (Qiagen). For each sample, 0.5 *μ*g of total RNA was reverse transcribed to cDNA using random hexamer primers (Invitrogen) and Superscript III reverse transcriptase (Invitrogen), following the manufacturer's instructions.

### Quantitative RT-PCR assay for time-series analyses of gene expression

cDNA samples were diluted 1:50 and amplified with the DyNAmo Flash SYBR Green qPCR Kit (Finnzymes) on a MyiQ Real-Time PCR Detection System (Bio-Rad). The two-step cycling program was as follows: 95°C for 7 min, followed by 40 cycles at 95°C for 15 s and 60°C for 1 min. Melt curve analyses were performed after each amplification to ensure specificity of products. Each cDNA sample was run in duplicates on separate plates. PCR efficiencies were calculated for each amplification with the software LinRegPCR [82]. Any wells showing strongly deviating PCR efficiencies of either target or reference genes were excluded from further analysis. We used transcription level measurements for the *P. patens *beta-tubulin 1 gene (Phypa_186458), as a reference to normalize target gene transcription levels. The Pp*TUB1 *reference gene displayed consistent amplification over all sample time points and treatments without evidence of circadian rhythmicity (Additional file 5). The CT values of duplicates were averaged, and the difference of the mean CT values for reference and target genes (ΔCT) was calculated for each sample time point and treatment combination. Target and reference primer sequences are listed in Additional file 6. The software COSOPT was used to detect significant circadian expression patterns under constant conditions (DD), under all temperatures, among the genes in the *P. patens *time series data [83-85]. In addition, a Fisher's exact g test, as implemented in the statistical software R package GeneCycle http://cran.r-project.org/web/packages/GeneCycle/index.html, was used to search for any harmonic pattern with unknown frequency in the time series expression data measured under constant conditions [86,87]. Comparative expression data for *Arabidopsis *measured under diurnal and circadian conditions were downloaded from the DIURNAL database [[88], http://diurnal.cgrb.oregonstate.edu/].

### Sequence analyses and reconstruction of phylogenetic relationships

Searches for homologs of *Arabidopsis *circadian clock components in other plant genomes used the following releases: *Physcomitrella patens *v1.1 [http://www.cosmoss.org/; [89]], *Oryza sativa *http://riceblast.dna.affrc.go.jp, *Selaginella moellendorffii *v1.0 http://genome.jgi-psf.org/Selmo1/Selmo1.home.html, and *Chlamydomonas reinhardtii *v4.0 [http://genome.jgi-psf.org/Chlre4/Chlre4.home.html; [90]]. Search criteria included sequences from the above databases with e-values ≤ 1e^-10 ^and a domain structure similar to the one found in Arabidopsis proteins. Conserved regions of predicted protein sequences were aligned with the software MUSCLE [91]. Protein sequence phylogenies were analyzed with a maximum likelihood model as implemented in PHYML 3.0 [92] and with a Bayesian inference method as implemented in MrBayes 3.1 [93,94]. The SMART database http://smart.embl-heidelberg.de/ was used for analyses of annotated protein domains and motifs [95,96]. Test for positive selection using dN/dS (*ω*) ratios were performed on an alignment of selected *PRR *genes from angiosperms (Additional file 3). We used the branch-site test of positive selection as implemented in PAML 4.3 [97]. Briefly, the branches of the tree are divided into foreground (the one leading to the *TOC1 *clade) and background branches (all other branches). Codon sites are separated into four classes with proportions *p*_*0*_*, p*_*1*_*, p*_*2a *_and *p*_*2b*_. Class 0 have *ω *< 1, and class 1 *ω *= 1 on both types of branches. Class 2*a *has *ω *< 1 on the background branch and *ω *≥ 1 on the foreground branch. Finally, class 2*b *has *ω *= 1 on the background branch and *ω *≥ 1 on the foreground branch. The test for positive selection is then obtained by comparing the likelihood of this model to the same model except that *ω *is fixed to 1 in site classes 2*a *and 2*b *on the foreground branch [97]. Twice the log likelihood difference between the two compared models (*2Δl)*, was compared against *χ*^*2 *^with 1 df.

## Authors' contributions

KH and UL conceived and designed the experiments and wrote the paper. KH carried out bioinformatics searches, phylogenetic reconstructions and the statistical analyses of time series data. TK carried out the PAML analysis. KH, TK, NG and HH carried out the experiments. All authors read, edited and approved the final manuscript.

## Supplementary Material

Additional file 1 **Comparative overview of photoperiodic pathway components associated to clock function in Arabidopsis and *P. patens***. Comparative overview of photoperiodic pathway components associated to clock function in *A. thaliana *and putative orthologs in *P. patens*, followed by the number of putative orthologs in the red alga *Cyanidioschyzon merolae*, the green algae *Ostreococcus tauri *and *Chlamydomonas reinhardtii *and the non-seed vascular plant *Selaginella moellendorffii*. Click here for file

Additional file 2 **Statistical analyses of circadian rhythms of gene expression data**. Statistical analyses of circadian rhythms in constant darkness (DD) at different temperatures. Click here for file

Additional file 3 **Test for positive selection**. Sequence data and phylogeny used for test of positive selection on *PRR *genes using PAML 4.3. Likelihood values and parameter estimates. Click here for file

Additional file 4 **Period length estimates of gene expression data in different temperatures**. Period lengths estimated with COSOPT plotted against temperature for putative clock genes in *P. patens*. Click here for file

Additional file 5 **Test of reference gene (*PpTUB1*) stability**. Experimental procedure for measurement of cDNA concentration in all time series and plots of *PpTUB1 *expression stability over time. Click here for file

Additional file 6 **Quantitative RT-PCR primer sequences and time series plots**. Names and sequences of primers used in the assay of gene expression and time series plots of gene expression levels including standard deviation from duplicate runs. Click here for file
