# Supplementary material for: Does the core circadian clock in the moss Physcomitrella patens (Bryophyta) comprise a single loop?
Source: BMC Plant Biol. 2010 Jun 15;10:109. doi: 10.1186/1471-2229-10-109 (PMC3017809; doi:10.1186/1471-2229-10-109)
Supplement: Additional file 1 — Comparative overview of photoperiodic pathway components associated to clock function in Arabidopsis and P. patens. Comparative overview of photoperiodic pathway components associated to clock function in A. thaliana and putative orthologs in P. patens, followed by the number of putative orthologs in the red alga Cyanidioschyzon merolae, the green algae Ostreococcus tauri and Chlamydomonas reinhardtii and the non-seed vascular plant Selaginella moellendorffii. [file 1471-2229-10-109-S1.DOC]

**Additional file 1**.

Comparative overview of photoperiodic pathway components associated to clock function in *A. thaliana* and putative orthologs in *P. patens*, followed by the number of putative orthologs in the red alga *Cyanidioschyzon merolae*, the green algae *Ostreococcus tauri* and *Chlamydomonas reinhardtii* and the non-seed vascular plant *Selaginella moellendorffii*^a^.

| *Arabidopsis thaliana* | |  | *Physcomitrella patens* | |  | *C. merolae* | *O. tauri* | *C. reinhardtii* | *S. moellendorffii* |
| --- | --- | --- | --- | --- | --- | --- | --- | --- | --- |
| Gene | Locus ID | Protein features | Gene / gene model^b^ | Locus tag | Reference | Number of putative orthologs | |  |  |
| *PHYA*  *PHYB*  *PHYC*  *PHYD*  *PHYE* | At1g09570  At2g18790  At5g35840  At4g16250  At4g18130 | PAS_2, GAF, Phytochrome, PAS, HisKA, HATPase_c | *PpPHY1*  *PpPHY2*  *PpPHY3*  *PpPHY4*  *PpPHY5a/b3/c* | Phypa_222399  Phypa_225644  Phypa_185248  Phypa_218861  Phypa_208532/  Phypa_165601/  Phypa_115388 | Rensing *et al.* 2008. | 0 | 0 | 0 | 1 |
| *CRY1*  *CRY2* | At4g08920  At1g04400 | DNA_photolyase, FAD_binding_7 | *PpCRY1a*  *PpCRY1b* | Phypa_111603  Phypa_228718 | Rensing *et al*. 2008. | 4 | 3 | 4 | 5 |
| *PIF3*  *PIL1* | At1g09530  At2g46970 | Helix loop helix domain |  | Phypa_164347  Phypa_164436  Phypa_130842  Phypa_49637 |  | 0 | 0 | 0 | 3 |
| *DET1* | At4g10180 | DET1 multidomain (pfam09737) |  | Phypa_195255  Phypa_60947 |  | 0 | 1 | 0 | 1 |
| *COP1* | At2g32950 | RING, 7xWD40 |  | Phypa_167057  Phypa_233494  Phypa_114861  Phypa_185297  Phypa_223602  Phypa_139137  Phypa_168551  Phypa_205172  Phypa_121847 |  | 1 | 2  (RING + 2xWD40) | 1 | 1 |
| *FHY1* | At2g37678 | Unknown | NA | 0 |  | 0 | 0 | 0 | 0 |
| *FHY3* | At3g22170 | FAR1 DNA-binding, MULE transposase, ZnF_PMZ | NA | 0 |  | 0 | 0 | 0 | 0 |
| *SPA1* | At2g46340 | STYKc protein kinase, WD40 |  | Phypa_178433  Phypa_126406 |  | 0 | 0 | 0 | 1 |
| *SRR1* | At5g59560 | SRR1 superfamily (pfam07985) |  | Phypa_136455  Phypa_173476 |  | 0 | 1 | 1 | 1 |
| *CKB3*  *CKB4* | At3g60250  At2g44680 | CK_II_beta regulatory subunit |  | Phypa_219176  Phypa_59578  Phypa_116155  Phypa_75590 |  | 1 | 2 | 0 | 3 |
| *LHY*  *CCA1* | At1g01060  At2g46830 | Single Myb | *PpCCA1a*  *PpCCA1b* | Phypa_9003  Phypa_132382 | Rensing *et al.* 2008; Okada *et al.* 2009;  Corellou et al. 2009. | 5^c^ | 3^c^ | 2^c^ | 2 |
| *TOC1/*  *PRR1* | At5g61380 | Pseudo-response regulator, CCT | NA | 0 |  | 0 | 0 | 0 | 1(2) |
| *PRR9*  *PRR7*  *PRR5*  *PRR3* | At2g46790  At5g24470  At5g02810  At5g60100 | Pseudo-response regulator, CCT | *PpPRR1-4* | Phypa_154145  Phypa_165025  Phypa_173125  Phypa_165029 | Rensing *et al*. 2008.  Corellou et al. 2009. | 0 | 1 | 1^d^ | 2(4) |
| *GI* | At1g22770 | Unknown | NA | 0 |  | 0 | 0 | 0 | 2 |
| *LUX (PCL1)* | At3g46640 | Single Myb (GARP) | *PpLUX-L* | Phypa_47310 |  | 1 | 2 | 2 | 1 |
| *ELF4* | At2g40080 | DUF1313 | *PpELF4-L* | Phypa_49622 |  | 0 | 0 | 1 | 2 |
| *ELF3* | At2g25930 | Not known | *PpELF3-L1*  *PpELF-L2* | Phypa_66647  Phypa_165364 |  | 0 | 0 | 0 | 3 |
| *ZTL*  *LKP2*  *FKF1* | At5g57360  At2g18915  At1g68050 | PAS/PAC, F-box, Kelch repeats | NA | 0 |  | 0 | 0 | 0 | 2 |
| *ARR3*  *ARR4* | At1G59940  At1G10470 | Response regulator |  | Phypa_27364  Phypa_18603  Phypa_17862  Phypa_47477  Phypa-47455  Phypa_36019  Phypa_35913 |  | 0 | 1 | 0 | 4 |
| *TIC* | At3G22380 | Unknown | NA | 0 |  | 0 | 0 | 0 | 0 |
| *TEJ* | At2g31870 | PARG |  | Phypa_115347  Phypa_13544 |  | 0 | 0 | 0 | 2 |
| *FIO1* | At2g21070 | Metyltransfer_10 |  | Phypa_152340 |  | 0 | 1 | 1 | 1 |
| *XCT* | At2g21150 | Colied coil, XAP5 |  | Phypa_132717  Phypa_141817 |  | 0 | 0 | 1 | 1 |
| *CCR2* | At2g21660 | RRM domain |  | Phypa_19797  Phypa_73609  Phypa_16354  Phypa_136379  Phypa_118845 |  | 1 | 2 | 4 | 3 |
| *EPR1*  *RVE2* | At1g18330  At5g37260 | Single Myb |  | Phypa_24905  Phypa_90560  Phypa_87648 |  | 5^c^ | 3^c^ | 2^c^ | 2 |

^a^ The following genome databases were used for BLAST searches: *Physcomitrella patens* (http://www.cosmoss.org/), *Cyanidioschyzon merolae* (http://merolae.biol.s.u-tokyo.ac.jp), *Ostreococcus tauri*, *Chlamydomonas reinhardtii* and *Selaginella moellendorffii* (http://genome.jgi-psf.org). Search criteria included a threshold e-value ≤ 1e-10, and an analogous domain structure to the Arabidopsis reference protein for significant pair-wise alignments to be counted as hits. Phylogenetic relationships between groups of putative orthologs and the Arabidopsis reference proteins were reconstructed with the software PHYML 3.0 (Guindon and Gascuel, 2003).

^b^ *P. patens* gene or gene model within groups does not necessarily correspond to *A. thaliana* gene listed on the same row.

^c^ We were not able to separate the closely related Myb-like domain of LHY/CCA1-, EPR1- and RVE2-like orthologs in *C. merolae*, *O. tauri* and *C. reinhardtii*; thus, the number of putative hits in these columns refer to the same loci.

^d^ Corellou *et al*. 2009, refer to an as yet unidentified TOC1 homolog in *C. reinhardtii* with transcript reference C_530090. This sequence corresponds to protein id Chlre2-166745, which contains a CCT motif but lacks a receiver domain at the N-terminal and is therefore an unlikely TOC1/PRR-related gene. The putative protein we have identified and refer to here is Chlre4-166515, which does contain the appropriate domains and display sequence similarity to the TOC1/PRR family of genes. See figure 2B and text for details.
