# Supplementary material for: Does the core circadian clock in the moss Physcomitrella patens (Bryophyta) comprise a single loop?
Source: BMC Plant Biol. 2010 Jun 15;10:109. doi: 10.1186/1471-2229-10-109 (PMC3017809; doi:10.1186/1471-2229-10-109)
Supplement: Additional file 2 — Statistical analyses of circadian rhythms of gene expression data. Statistical analyses of circadian rhythms in constant darkness (DD) at different temperatures. [file 1471-2229-10-109-S2.DOC]

**Additional file 2**. Statistical analysis of circadian rhythms in constant darkness (DD) at different temperatures.

| COSOPT |  |  |  | Fisher’s exact  g test |
| --- | --- | --- | --- | --- |
| Condition | GeneID | Period (h) | pMMC-β^a^ | p-value |
| DD, 17°C | PpCCA1a | 21.2 | 0.026* | 0.006* |
|  | PpCCA1b | 21.9 | 0.030* | 0.007* |
|  | PpPRR1 | 21.2 | 0.019* | 0.005* |
|  | PpPRR2 | 21.5 | 0.042* | 0.000** |
|  | PpPRR3 | 20.9 | 0.050* | 0.016* |
|  | PpPRR4 | 21.5 | 0.030* | 0.178 |
|  | PpELF3-L1 | 22.5 | 0.025* | 0.017* |
|  | PpELF3-L2 | 20.8 | 0.160 | 0.090 |
|  | Phypa_233510 | 22.5 | 0.806 | 0.102 |
|  | Phypa_49622 | 25.8 | 0.187 | 0.269 |
|  | Phypa_47310 | 23.7 | 0.238 | 0.072 |
|  | Phypa_25200 | 10.7 | 0.858 | 0.128 |
|  | Phypa_34062 | 36.8 | 0.717 | 0.837 |
|  | Phypa_34063 | 26.9 | 0.346 | 0.303 |
| DD, 12°C | PpCCA1a | 21.5 | 0.019* | 0.011* |
|  | PpCCA1b | 22.5 | 0.017* | 0.085 |
|  | PpPRR1 | 24.2 | 0.589 | 0.235 |
|  | PpPRR2 | 16.9 | 0.140 | 0.544 |
|  | PpPRR3 | 25.4 | 0.254 | 0.236 |
|  | PpPRR4 | 21.4 | 0.992 | 0.078 |
|  | PpELF3-L1 | 21.6 | 0.103 | 0.241 |
|  | PpELF3-L2 | 23.5 | 0.167 | 0.202 |
|  | Phypa_49622 | 24.8 | 0.149 | 0.481 |
|  | Phypa_47310 | 10.7 | 0.240 | 0.885 |
| DD, 25°C | PpCCA1a | 21.9 | 0.080 | 0.006* |
|  | PpCCA1b | 22.4 | 0.013* | 0.000** |
|  | PpPRR1 | 23.2 | 0.032* | 0.101 |
|  | PpPRR2 | 24.5 | 0.042* | 0.035 |
|  | PpPRR3 | 25.1 | 0.169 | 0.0562 |
|  | PpPRR4 | 22.9 | 0.298 | 0.543 |
|  | PpELF3-L1 | 20.0 | 0.062 | 0.293 |
|  | PpELF3-L2 | 21.2 | 0.475 | 0.148 |
|  | Phypa_49622 | 30.6 | 0.321 | 0.370 |
|  | Phypa_47310 | 12.5 | 0.303 | 0.580 |

^a^ qPCR data from 48 h in DD were analyzed with the software COSOPT to estimate period length and the probability that the cosine curve with the best fit to the data has a significant amplitude with a cutoff value of pMMC-β < 0.05 (Straume, M. 2004; Edwards, K.D. *et al*. 2006).
