# Supplementary material for: Does the core circadian clock in the moss Physcomitrella patens (Bryophyta) comprise a single loop?
Source: BMC Plant Biol. 2010 Jun 15;10:109. doi: 10.1186/1471-2229-10-109 (PMC3017809; doi:10.1186/1471-2229-10-109)
Supplement: Additional file 3 — Test for positive selection. Sequence data and phylogeny used for test of positive selection on PRR genes using PAML 4.3. Likelihood values and parameter estimates. [file 1471-2229-10-109-S3.DOCX]

**Additional file 3**

I. Sequences included in PAML analysis.

| **Gene** | **GI** | **Database** |
| --- | --- | --- |
| APRR3 | NM_125403 | NCBI |
| APRR5 | NM_122355 | NCBI |
| APRR7 | AB046954 | NCBI |
| APRR9 | NM_130245 | NCBI |
| CsTOC1 | AY611028 | NCBI |
| LgPRR37 | AB243684 | NCBI |
| LgPRR59 | AB243685 | NCBI |
| LgPRR95 | AB243686 | NCBI |
| McTOC1 | AY371288 | NCBI |
| OsPRR1 | AB189038 | NCBI |
| OsPRR37 | AB189042 | NCBI |
| OsPRR59 | AK120059 | KOME rice database |
| OsPRR73 | AB189040 | NCBI |
| OsPRR95 | AB189041 | NCBI |
| PtPRR3 | XM_002321313 | NCBI |
| PtPRR5 | XM_002320196 | NCBI |
| PtPRR6 | XM_002301407 | NCBI |
| PtPRR7 | XM_002311088 | NCBI |
| PtPRR9 | XM_002330094 | NCBI |
| PtPRRA | XM_002318466 | NCBI |
| PtPRRB | XM_002316297 | NCBI |
| Rc_1 | XM_002514679 | NCBI |
| Rc_2 | XM_002515033 | NCBI |
| Rc_3 | XM_002524383 | NCBI |
| Rc_4 | XM_002525153 | NCBI |
| Rc_5 | XM_002531790 | NCBI |
| SmTOC1 | Selmo1_438647 | JGI |
| TOC1 | NM_125531 | NCBI |
| ZmPRR73 | EU952116 | NCBI |
| ZmPRR95 | NM_001158064 | NCBI |
| ZmTOC1 | NM_001154351 | NCBI |


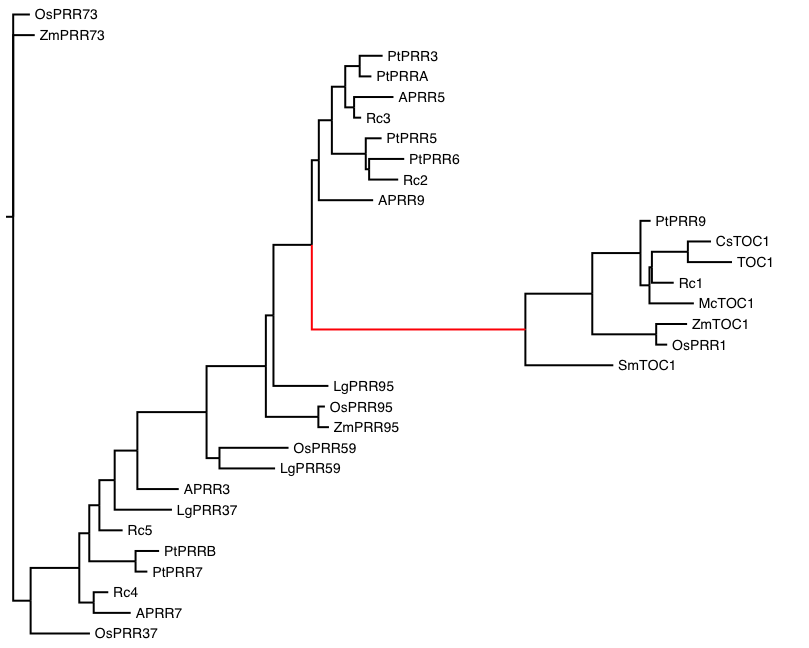


II. Un-rooted phylogeny for the *TOC1/PRR* family in vascular plants. Test for positive selection was done on the branch separating the *PRR* subfamily from the *TOC1*-like subfamily (indicated in red).

III. Likelihood values and parameter estimates under branch-site model A, with *ω_2_* fixed (null model) and branch-site model A, with *ω_2_* not fixed.

| **Model** | **Likelihood** | **Proportion of site classes** | | | **Foreground *ω*** |
| --- | --- | --- | --- | --- | --- |
|  |  | ***p_0_*** | ***p_1_*** | ***p_2a_ + p_2b_*** |  |
| Branch-site model A, with *ω_2_* = 1 fixed | -8378.656 | 0.726 | 0.068 | 0.206 | 1 |
| Branch-site model A, with *ω_2_* > 1 | -8375.278 | 0.714 | 0.068 | 0.219 | 5.5 |
