# Supplementary material for: Does the core circadian clock in the moss Physcomitrella patens (Bryophyta) comprise a single loop?
Source: BMC Plant Biol. 2010 Jun 15;10:109. doi: 10.1186/1471-2229-10-109 (PMC3017809; doi:10.1186/1471-2229-10-109)

Additional file 4. COSOPT period length estimates plotted against temperature for putative clock genes in *P. patens*.

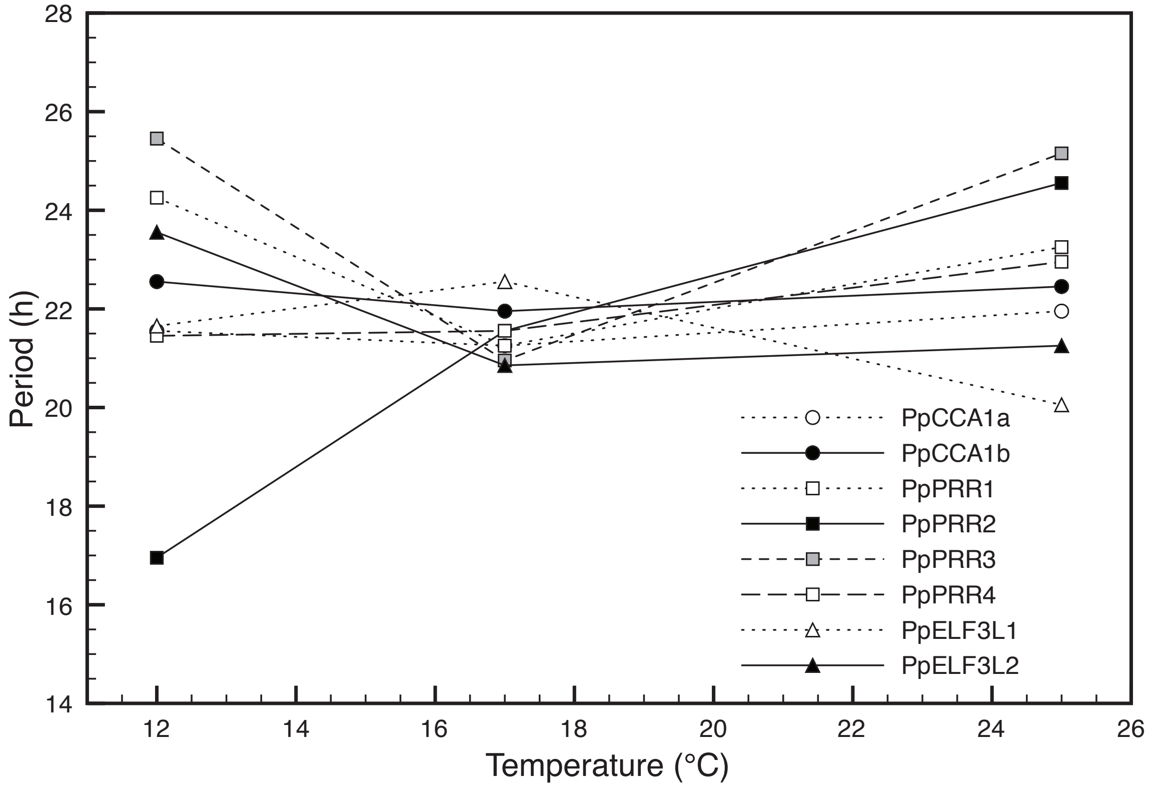

Supplement: Additional file 4 — Period length estimates of gene expression data in different temperatures. Period lengths estimated with COSOPT plotted against temperature for putative clock genes in P. patens. [file 1471-2229-10-109-S4.PDF]
