# Supplementary material for: Does the core circadian clock in the moss Physcomitrella patens (Bryophyta) comprise a single loop?
Source: BMC Plant Biol. 2010 Jun 15;10:109. doi: 10.1186/1471-2229-10-109 (PMC3017809; doi:10.1186/1471-2229-10-109)
Supplement: Additional file 5 — Test of reference gene (PpTUB1) stability. Experimental procedure for measurement of cDNA concentration in all time series and plots of PpTUB1 expression stability over time. [file 1471-2229-10-109-S5.DOCX]

**Additional file 5.** Test of reference gene (Pp*TUB1*) stability.

To test the temporal stability of our reference gene Pp*TUB1*, we implemented a recent method to quantify cDNA concentration based on OliGreen (Invitrogen). OliGreen is a dye that emits fluorescence when bound to single stranded nucleic acids (Rhinn *et al*. 2008a) but is insensitive to free nucleotides and to very short oligonucleotides. If measurements are conducted at 80°C, the influence of RNA in the sample is negligible (Rhinn *et al*. 2008b).

cDNA was synthesized from each RNA sample included in the study, and used to quantify cDNA and Pp*TUB1* concentration. Duplicates of two µl of cDNA were mixed with 23 µl of a 1:200 dilution of OliGreen, and fluorescence was measured during one minute at 80°C using the SybrGreen channel on a MyiQ Real-Time PCR Detection System (Bio-Rad). 1:50 dilutions of the same cDNA samples were also used to quantify Pp*TUB1* following our standard protocol.

Pp*TUB1* CT values adjusted for cDNA concentration showed limited variation over time, with CV below 3 %, and without clear evidence for circadian rhythm (figure 1-3). To test if weak rhythmic Pp*TUB1* expression still could affect interpretation of the expression patterns for putative clock genes, data for Pp*CCA1a* was plotted using raw CT values, together with values adjusted with Pp*TUB1* and cDNA concentration as measured with OliGreen. As seen from figure 4, patterns obtain from the different estimates of Pp*CCA1a* all revealed similar rhythmic expression patterns.


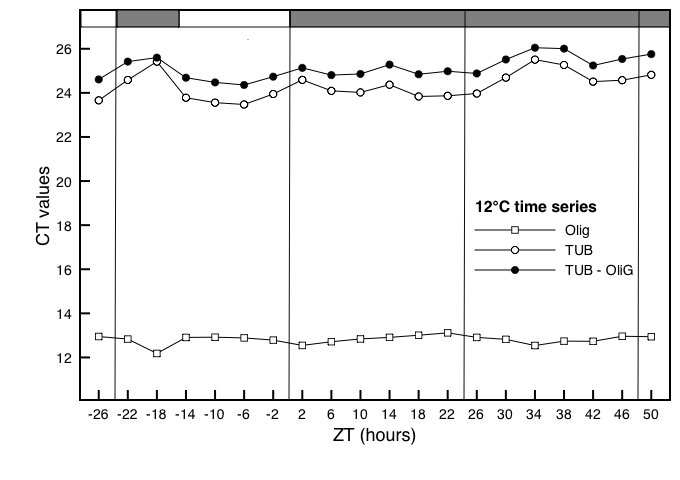


Figure 1. Stability of Pp*TUB1* in the 12°C experiment. CT values for Pp*TUB1* plotted together with log2 values of OliGreen fluorescence and Pp*TUB1* values adjusted for cDNA concentration. Adjustment was made by subtracting –log2 values of OliGreen fluorescence and adding a constant to put estimates on a similar scale. OliG, 2log OliGreen fluorescence; TUB, CT Pp*TUB1*; TUB – OliG, CT Pp*TUB1* – (-log2 OliGreen fluorescence) + constant.


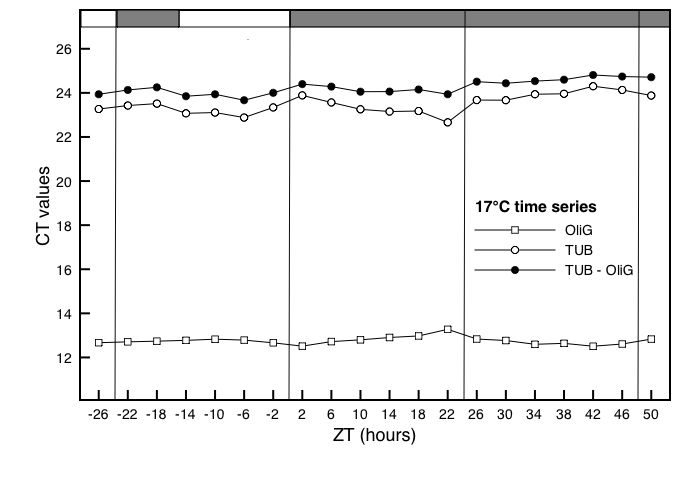


Figure 2. Stability of Pp*TUB1* in the 17°C experiment. See figure 1 for details.


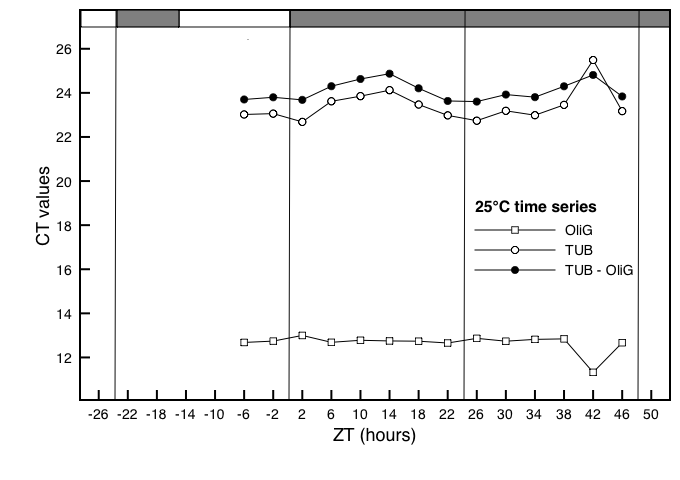


Figure 3. Stability of Pp*TUB1* in the 25°C experiment. See figure 1 for details.


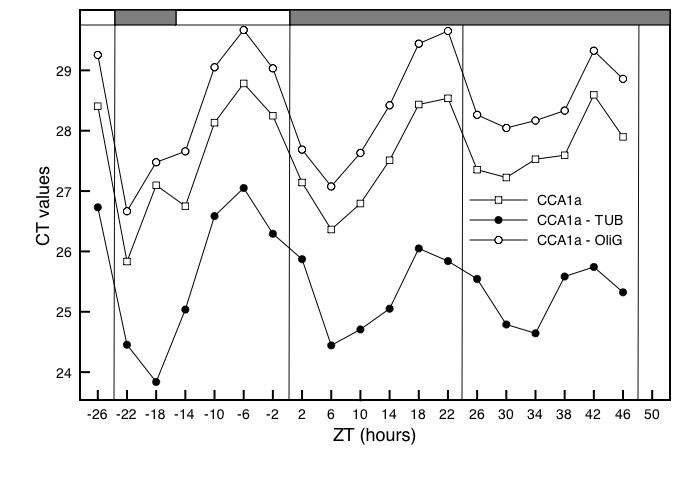


Figure 4. The effect of normalization method on Pp*CCA1a* expression. CT values for Pp*CCA1a* plotted together with estimated of Pp*CCA1a* expression adjusted with Pp*TUB1* CT values and those adjusted with log2 OliGreen estimates. OliGreen adjustments were made as in figure 1.

References

Rhinn H, Scherman D, Escriou V: **One-step quantification of single-stranded DNA in the presence of RNA using Oligreen in a real-time polymerase chain reaction thermocycler**. Analytical Biochemistry 2008a, 372:116.

Rhinn H, Marchand-Leroux C, Croci N, Plotkine M, Scherman D, Escriou V: **Housekeeping while brain's storming Validation of normalizing factors for gene expression studies in a murine model of traumatic brain injury.** BMC Mol Biol 2008b 9:62.
