# Supplementary material for: Does the core circadian clock in the moss Physcomitrella patens (Bryophyta) comprise a single loop?
Source: BMC Plant Biol. 2010 Jun 15;10:109. doi: 10.1186/1471-2229-10-109 (PMC3017809; doi:10.1186/1471-2229-10-109)

Additional file 6

I. Primer sequences used for qPCR amplification.

Target	Forward (5’ -> 3’)	Reverse (5’ -> 3’)
PpCCA1a (Phypa_9003)	AGAGCTTCAATGCCTCCAAA	AAGGCACAGGTACCAAGGTG
PpCCA1b (Phypa_132382)	TGAGGGGTGTTTCTACGGAAGAC	TCGGTCAAAGCAAAAGTCAAGC
PpPRR1 (Phypa_154145)	TGATGAAACGGGAGGCTTGC	AAAGGCAGCGGAACACAATG
PpPRR2 (Phypa_165025)	GCTCTCCTACGGAATGCAACTATG	TTCCAACAGCCCCCATGCTAAG
PpPRR3 (Phypa_173125)	TTCTCAAGGGATGGGTGCCAAC	GCTTTGCGGTATCACAAGTAGACC
PpPRR4 (Phypa_165029)	CGAAGATGATGAAGCGGGAAG	GGAACACGATGCCAAGACTGTC
PpLUXL (Phypa_47310)	ATCCCTTGTTCTGGCATCTG	GGTAGTGCCTCGAACGACAT
Phypa_34063	AGCCTGCCTACAGGTGAAGA	ACTCGCAGGTCAAGCAAACT
Phypa_34062	GCGAAGAATTGACTCCCTCA	GCTAGCATGGGATTCTCGAC
Phypa_25200	GCTGGTAAGGTGCCAGAGAG	CTTTCGCCCGAAGTCTGTAG
PpELF4L (Phypa_49622)	GGCGAGTGTCTTCCATCTTCAAC	GAATCTTGGCTTCGTGGTTCTG
PpELF3-L1 (Phypa_66647)	TGATGCAACAATGTGGTGTG	GCAGCTGATTCTTGGGTAGC
PpELF3-L2 (Phypa_165364)	GTTCTCAACCCTTGCAGCTC	GTGATATCCTTGCGGCGTAT
Phypa_233510	CCACAGCTAGTGGGGAATGT	CACAAAACCACCGTCACAAG
PpTUB1 (Phypa_186458)	GACTGCTTGCAAGGTTTCCAAG	GTTCAAGTCGCCAAACGAAGGA

II. Normalized expression levels with error bars denoting the standard deviation from duplicate qPCR runs on separate plates.

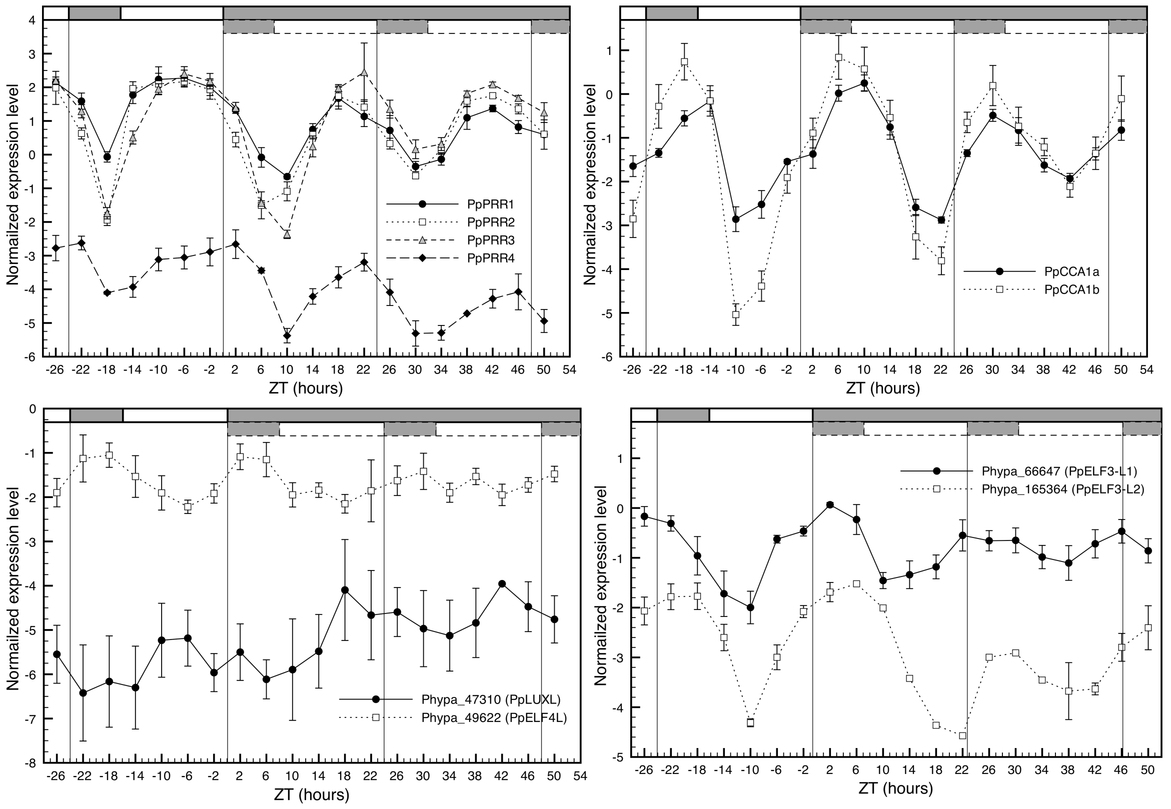

Supplement: Additional file 6 — Quantitative RT-PCR primer sequences and time series plots. Names and sequences of primers used in the assay of gene expression and time series plots of gene expression levels including standard deviation from duplicate runs. [file 1471-2229-10-109-S6.PDF]
